# Endogenous CNS Expression of Neurotensin and Neurotensin Receptors Is Altered during the Postpartum Period in Outbred Mice

**DOI:** 10.1371/journal.pone.0083098

**Published:** 2014-01-08

**Authors:** Terri M. Driessen, Changjiu Zhao, Anna Whittlinger, Horecia Williams, Stephen C. Gammie

**Affiliations:** 1 Department of Zoology, University of Wisconsin-Madison, Madison, Wisconsin, United States of America; 2 Department of Animal Science, Fort Valley State University, Fort Valley, Georgia, United States of America; 3 Neuroscience Training Program, University of Wisconsin-Madison, Madison, Wisconsin, United States of America; Kent State University, United States of America

## Abstract

Neurotensin (NT) is a neuropeptide identical in mice and humans that is produced and released in many CNS regions associated with maternal behavior. NT has been linked to aspects of maternal care and previous studies have indirectly suggested that endogenous NT signaling is altered in the postpartum period. In the present study, we directly examine whether NT and its receptors exhibit altered gene expression in maternal relative to virgin outbred mice using real time quantitative PCR (qPCR) across multiple brain regions. We also examine NT protein levels using anti-NT antibodies and immunohistochemistry in specific brain regions. In the medial preoptic area (MPOA), which is critical for maternal behaviors, mRNA of NT and NT receptor 3 (Sort1) were significantly up-regulated in postpartum mice compared to virgins. NT mRNA was also elevated in postpartum females in the bed nucleus of the stria terminalis dorsal. However, in the lateral septum, NT mRNA was down-regulated in postpartum females. In the paraventricular nucleus of the hypothalamus (PVN), Ntsr1 expression was down-regulated in postpartum females. Neurotensin receptor 2 (Ntsr2) expression was not altered in any brain region tested. In terms of protein expression, NT immunohistochemistry results indicated that NT labeling was elevated in the postpartum brain in the MPOA, lateral hypothalamus, and two subregions of PVN. Together, these findings indicate that endogenous changes occur in NT and its receptors across multiple brain regions, and these likely support the emergence of some maternal behaviors.

## Introduction

In mammals, the transition from a virgin to a postpartum state is accompanied by a suite of physiological, sensory, and behavioral changes. Progesterone, estradiol, and prolactin release in late pregnancy and early postpartum, as well as sensory input from pups, have been shown to help facilitate the onset of maternal behaviors in rodents, such as pup retrieval, pup licking and grooming, nursing, and offspring protection [1], [2]. Hormonal changes and sensory input from offspring modulate the CNS in part by altering expression of critical maternal behavior genes. Postpartum expression changes have been identified in genes involved in oxytocin, dopamine, and opioid (enkephalin) signaling [3]–[8]. However, changes in other signaling systems that may support maternal care are still under-explored.

Recent studies have indicated that modulation of signaling of the neuropeptide, neurotensin (NT), may contribute to the maternal state. For example, in the medial preoptic area (MPOA), increased activity of NT positive neurons was found to be associated with elevated maternal profiles in mice [9]. Also, the electrophysiological response of oxytocin neurons to NT is altered in postpartum rats [10] and these oxytocin neurons are linked to maternal behaviors, including the milk ejection reflex [8], [11], [12]. Intracerebroventricular (icv) injections of NT suppress offspring protection, while antagonizing neurotensin receptor 1 (Ntsr1) elevates defense [13]. Further, immediate early gene activation is decreased in postpartum females compared to virgins after icv injection of NT [14], [15]. Thus, NT could have a complex action with it supporting some maternal behaviors in certain brain regions (e.g., MPOA) and suppressing other behaviors, such as offspring protection, in different regions.

Neurotensin is a highly conserved neuropeptide first isolated in bovine hypothalamus [16]. There are three known NT receptors, including Ntsr1 [17] and neurotensin receptor 2 (Ntsr2) [18], [19], which are both G-protein coupled receptors. The third NT receptor is termed sortilin 1 (Sort1) [20], and is a one-transmembrane domain sorting receptor found primarily within the cell [21]. NT and its receptors are found in a number of brain regions linked to maternal behavior, including the nucleus accumbens (NAcc), lateral septum (LS), bed nucleus of the stria terminalis, dorsal (BnSTd), MPOA, paraventricular nucleus (PVN), lateral hypothalamus (LH), central and basolateral amygdala (BLA/CeA), and ventral tegmental area (VTA) [22]–[28]. NT signaling is often linked to dopamine signaling in various regions [29], [30], and dopamine itself has been linked to maternal care [31]–[33]. In addition to modulating the activity of the hypothalamic pituitary adrenal (HPA) axis [34], [35], NT release and activation of NT receptors have been shown to affect temperature regulation [36], [37] and pain perception [38]. Further, NT has been linked to some mental health disorders, including schizophrenia and autism [39]–[41].

Postpartum females undergo a number of experiences, including pregnancy, parturition, lactation, and pup exposure, that shape the maternal brain and facilitate maternal care [2], [42]. Although a number of studies have indirectly suggested the likelihood of altered expression of NT and its receptors in the postpartum CNS, to date, no study has directly examined this possibility. The goal of this study was to determine if the combined effects of pregnancy, parturition, lactation, and pup exposure are associated with altered NT and NT receptor expression compared to virgin counterparts. We used real time quantitative PCR (qPCR) and immunohistochemistry (IHC) using an anti-NT antibody to determine if endogenous NT and NT receptors are differentially regulated in the postpartum brain compared to virgin mice. Brain regions associated with maternal behaviors and/or NT signaling were evaluated.

## Results

### Changes in NT and NT receptor mRNA expression is Variable and Region Specific

#### MPOA

**Figure 1:**
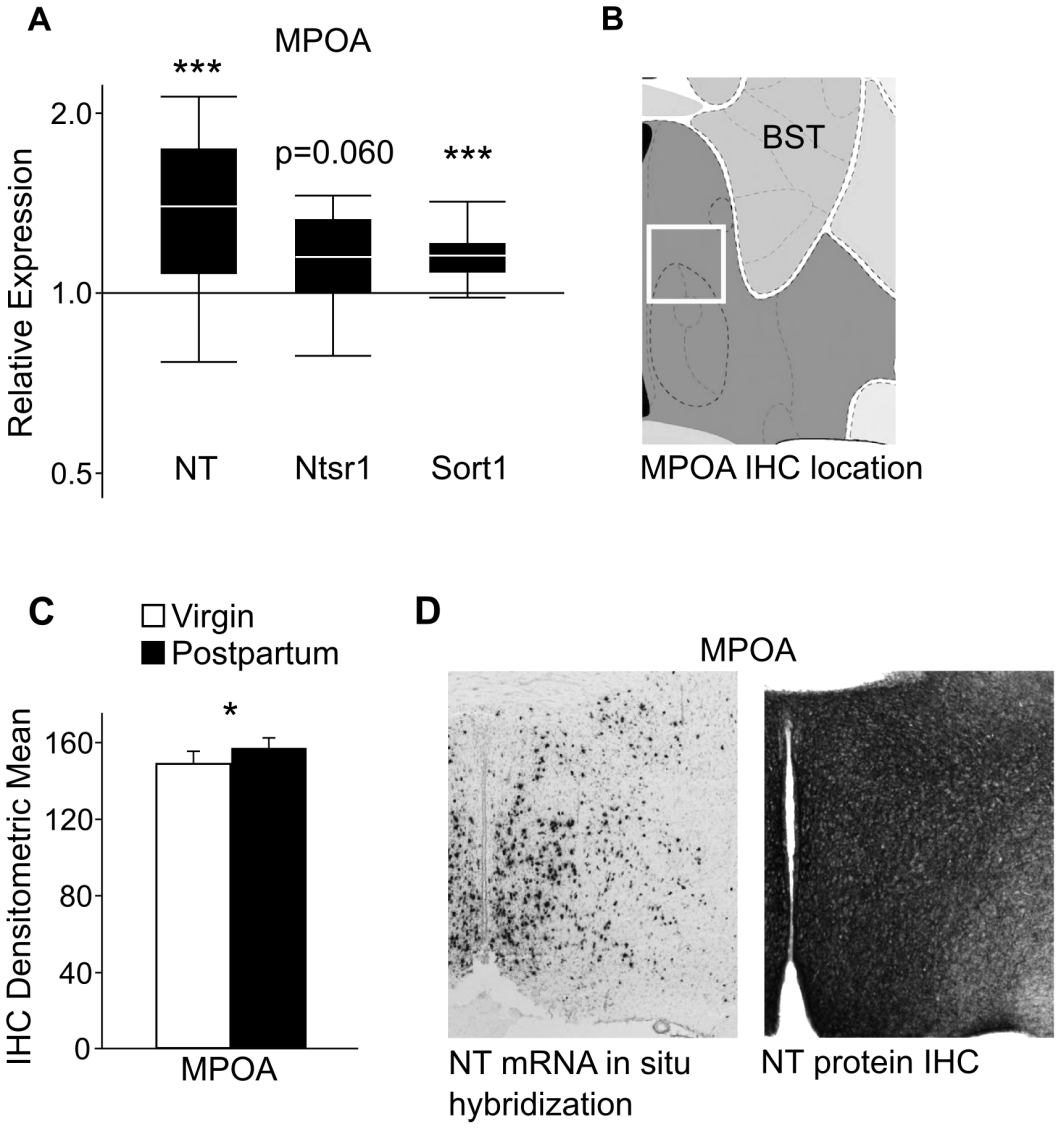
qPCR and IHC results for NT and NT receptor expression changes in the MPOA. (A) Relative expression (y-axis) represents a ratio of gene expression in postpartum versus virgin females, with ratios over one indicating genes that have higher expression in lactating females. The box whisker plots demarcate the range (whiskers), interquartile range (box), and median (solid white line) for each gene tested. qPCR revealed significant up-regulation of NT and Sort1 in the postpartum MPOA, with a nonsignificant increase in Ntsr1. Sample size: 6 virgin and 8 postpartum females. (B) The white box outlines the area of the MPOA examined for NT peptide expression. Image modified from the Allen Mouse Brain Atlas. (C) A one-way ANOVA revealed a significant elevation in the density of NT peptide labeling in the MPOA of postpartum versus virgin females. Sample size: 7 virgins and 9 postpartum females. (D) Many cell bodies in the ventral and medial portion of the MPOA contain NT mRNA (left panel). Image modified from the Allen Mouse Brain Atlas. Dense NT peptide labeling is found throughout the MPOA (right panel), suggesting that NT peptide may be transported into the MPOA from other regions. * p<0.05; *** p<0.005. BST = bed nucleus of stria terminals.

In the MPOA, NT mRNA was up-regulated in lactating mice compared to virgins (p = 0.023), as was Sort1 (p = 0.002) (Fig. 1A). There was also a statistically nonsignificant up-regulation of Ntsr1 in lactating females (p = 0.060) (Fig. 1A). No difference in expression was found in the MPOA for Ntsr2 (Table 1).

**Table 1.** Non-significant qPCR results and expression ratios for NT and NT receptors across all regions tested.

Region	Gene	Sample Number	Expression Ratio	P-value
NAcc	NT	Virgin = 11, Lactating = 11	1.05	0.671
	Ntsr1	Virgin = 11, Lactating = 11	1.08	0.229
	Ntsr2	Virgin = 11, Lactating = 11	1.07	0.515
	Sort1	Virgin = 11, Lactating = 11	0.95	0.244
LS	Ntsr1	Virgin = 10, Lactating = 11	1.05	0.508
	Ntsr2	Virgin = 10, Lactating = 11	0.97	0.537
BnSTd	Ntsr1	Virgin = 7, Lactating = 7	1.13	0.108
	Ntsr2	Virgin = 7, Lactating = 7	1.02	0.503
	Sort1	Virgin = 7, Lactating = 6	1.04	0.195
MPOA	Ntsr2	Virgin = 6, Lactating = 8	1.01	0.701
PVN	NT	Virgin = 7, Lactating = 7	1.11	0.351
	Ntsr2	Virgin = 7, Lactating = 7	1.03	0.603
	Sort1	Virgin = 7, Lactating = 7	1.04	0.211
LH	NT	Virgin = 11, Lactating = 12	1.05	0.497
	Ntsr1	Virgin = 10, Lactating = 12	0.96	0.415
	Ntsr2	Virgin = 10, Lactating = 12	0.99	0.843
	Sort1	Virgin = 11, Lactating = 12	1.04	0.224
BLA/CeA	NT	Virgin = 12, Lactating = 12	1.13	0.274
	Ntsr1	Virgin = 12, Lactating = 12	1.10	0.256
	Ntsr2	Virgin = 12, Lactating = 12	1.06	0.491
	Sort1	Virgin = 12, Lactating = 12	1.03	0.505
VTA	Ntsr1	Virgin = 7, Lactating = 7	0.90	0.565
	Ntsr2	Virgin = 7, Lactating = 7	0.96	0.377
	Sort1	Virgin = 7, Lactating = 7	1.03	0.707

Note: The sample numbers listed above were the final sample numbers used, with outliers removed, for data analysis. A complete list of original sample numbers for each brain region can be found in the Methods section. Outliers were removed if they were more than two standard deviations away from the group mean. The expression ratio compares the expression of the gene of interest in postpartum compared to virgin females. For values greater than 1, the gene is expressed at higher levels in the postpartum group compared to the virgin group. For values less than 1, the gene is expressed at lower levels in the postpartum group versus the virgin group.

#### PVN

**Figure 2:**
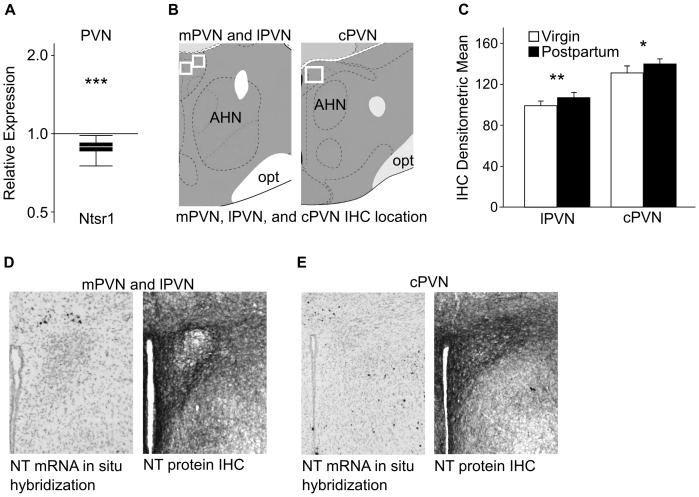
qPCR and IHC results for NT and NT receptor expression changes in the PVN. (A) Relative expression (y-axis) represents a ratio of gene expression in postpartum versus virgin females, with ratios under one indicating genes that have lower expression in lactating females. The box whisker plots demarcate the range (whiskers), interquartile range (box), and median (solid white line) for each gene tested. qPCR revealed significant down-regulation of Ntsr1 in postpartum versus virgin females. Sample size: 7 virgin and 7 postpartum females pooled from a total of 14 individuals for each group. (B) The white box outlines the areas of the PVN examined for NT peptide expression. Images modified from the Allen Mouse Brain Atlas. (C) A one-way ANOVA revealed a significant elevation in the density of NT peptide labeling in the lPVN and cPVN of postpartum versus virgin females. Sample size: 7 virgins and 8 postpartum females for lPVN, and 7 virgins and 7 postpartum females for cPVN. (D–E) Very few cell bodies that contain NT mRNA are found in certain subregions of the PVN (D and E, left panels). Images modified from the Allen Brain Atlas. NT peptide IHC revealed dense staining of fibers in the mPVN and cPVN (D and E, right panels), and moderate staining in the lPVN (D, right panel). This suggests that NT peptide is transported into the PVN for release. * p<0.05; ** p<0.01; *** p<0.005. AHN = anterior hypothalamic nucleus, opt = optic tract, lPVN = lateral PVN, mPVN = medial PVN, cPVN = caudal PVN.

Ntsr1 mRNA was down-regulated in postpartum versus virgin females in the PVN (p<0.001) (Fig. 2A). No changes in NT, Ntsr2, or Sort1 were found in the PVN (Table 1).

#### LS

**Figure 3:**
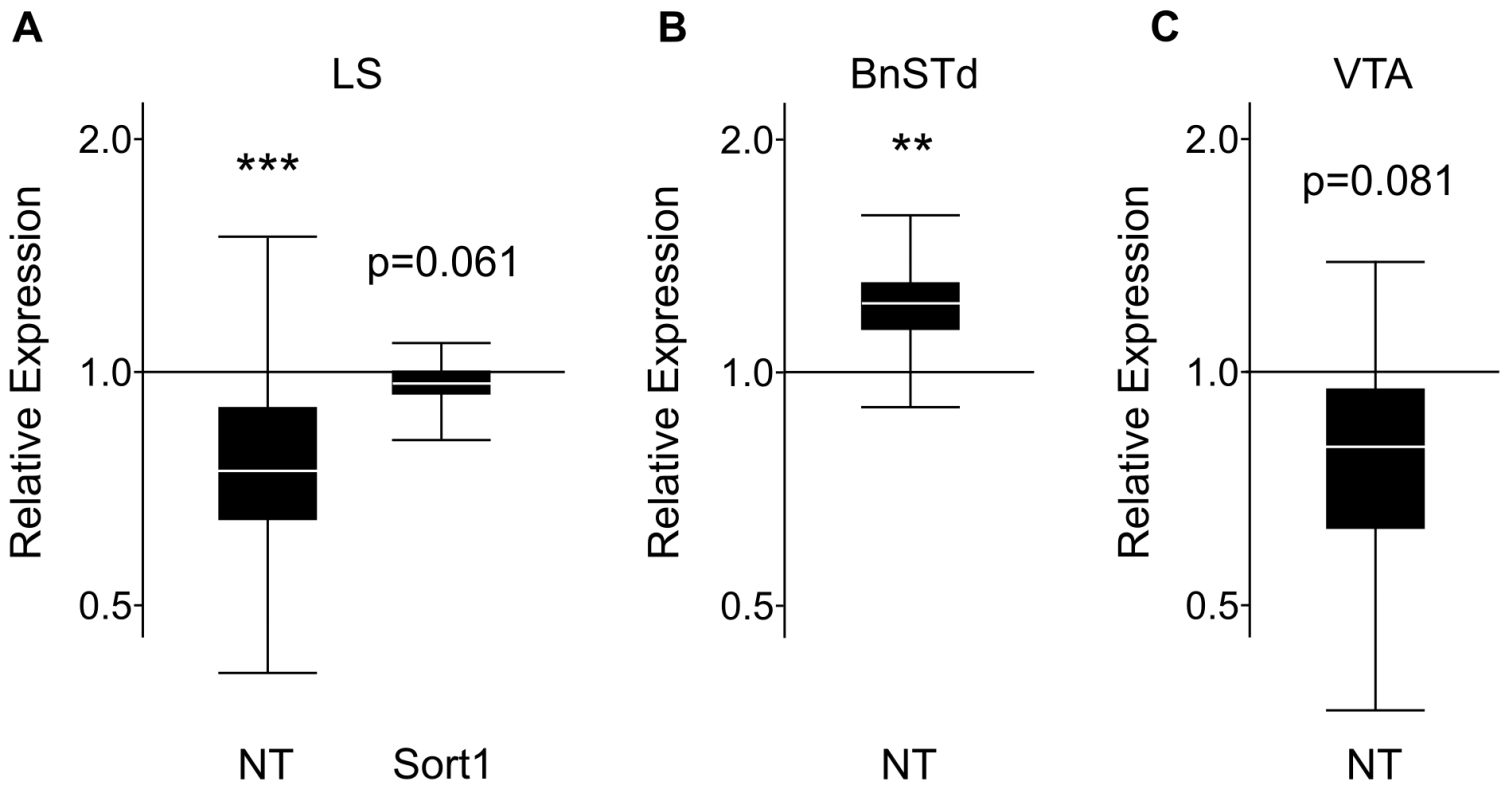
Results of qPCR analysis of NT and NT receptors in the LS, BnSTd, and VTA. Relative expression (y-axis) represents a ratio of gene expression in postpartum versus virgin females, with ratios under one indicating genes that have lower expression in lactating females. The box whisker plots demarcate the range (whiskers), interquartile range (box), and median (solid white line) for each gene tested. (A) In the LS, NT mRNA expression was significantly down-regulated in lactating females, and there was a nonsignificant decrease in Sort1 expression in postpartum females compared to virgin females. Sample size: 10 virgins and 11 postpartum females. (B) In the BnSTd, NT was significantly up-regulated in postpartum compared to virgin females. Sample size: 7 virgin and 7 postpartum females pooled from a total of 14 individuals from each group. (C) In the VTA, NT was nonsignificantly down-regulated in lactating females versus virgins. Sample size: 7 virgin and 7 postpartum females. * p<0.05; ** p<0.01; *** p<0.005.

Within the LS, NT mRNA expression was down-regulated in postpartum females (p = 0.003), and there was a statistically nonsignificant decrease in Sort1 mRNA (p = 0.061) (Fig. 3A). Ntsr1 and Ntsr2 expression did not change in the LS (Table 1).

#### BnSTd

In the BnSTd, NT expression was up-regulated in postpartum females compared to virgin females (p = 0.007) (Fig. 3B). NT receptor expression did not change in the BnSTd (Table 1).

#### NAcc, LH, BLA/CeA, and VTA

Within the VTA, there was a nonsignificant decrease in levels of NT mRNA in maternal females (p = 0.081) (Fig. 3C). No differences were observed for Ntsr1, Ntsr2 or Sort1 in the VTA (Table 1). In the NAcc, LH, and BLA/CeA, no differences in expression were found for NT or any of the three receptors (Table 1).

### NT Peptide Expression Increases in a Subset of Postpartum Brain Regions

IHC analysis showed a significant increase in NT labeling in the MPOA in lactating compared to virgin females (p = 0.021, one-way ANOVA) (Fig. 1C). One section in the virgin group appeared to have lighter staining than the rest of the sections within the group, but a Dixon test indicated that it was not an outlier, and the results were still significant when the section was omitted (p = 0.043). Staining was the most dense in this region, and the majority of staining was of fibers with some cell bodies. In situ hybridization for NT mRNA from the Allen Mouse Brain Atlas (http://mouse.brain-map.org) indicates that a number of NT positive neurons exist in the MPOA (Fig. 1D, left panel), so IHC labeling of NT could reflect NT from both local and external sources (Fig. 1D, right panel).

There was an increase in NT labeling in postpartum females in the lateral PVN (lPVN) (p = 0.005, one-way ANOVA), and caudal PVN (cPVN) (p = 0.011, one-way ANOVA) compared to virgin females (Fig. 2C). In situ hybridization for NT mRNA from the Allen Brain Atlas indicates that cell bodies containing NT are rare in the PVN subregions we analyzed using IHC (Fig. 2D–E). This suggests that NT is likely transported into the PVN from external sources.

**Figure 4:**
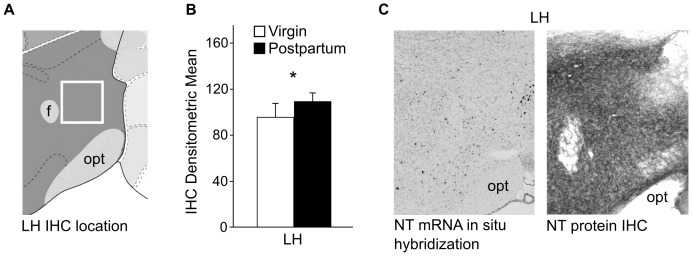
IHC results for changes in NT peptide expression in the LH. (A) The white box outlines the area of the LH examined for NT peptide expression. Image modified from the Allen Mouse Brain Atlas. (B) A one-way ANOVA revealed a significant elevation in the density of NT peptide labeling in the postpartum LH compared to virgin females. Sample size: 7 virgins and 8 postpartum females. (C) Few cell bodies that contain NT mRNA are found in the LH (left panel). Image modified from the Allen Brain Atlas. NT peptide IHC revealed dense staining of fibers in the LH (right panel). * p<0.05. f = fornix, opt = optic tract.

NT staining was increased in postpartum females in the LH compared to virgin females (p = 0.02, one-way ANOVA) (Fig. 4B). In situ hybridization from the Allen Brain Atlas indicated that some cell bodies contain NT mRNA (Fig. 4C, left panel). NT peptide IHC staining in this region was predominantly of fibers with very few cell bodies labeled (Fig. 4C, right panel). For all other regions, including NAcc, LS, BnSTd, CeA, and VTA, no differences in NT labeling were found (Table 2).

**Table 2:** Non-significant virgin and postpartum IHC mean density and one-way ANOVA results for each region or subregion tested.

Region	Virgin mean ± SEM (sample number)	Postpartum mean ± SEM (sample number)	One-way ANOVA results
NAcc	98.68±3.24 (7)	102.70±1.74 (8)	p = 0.278
rLSD	64.26±6.17 (6)	69.08±2.61 (8)	p = 0.699#
rLSV	108.37±4.09 (7)	108.52±2.43 (8)	p = 0.908#
mLSV	110.24±5.66 (7)	113.31±2.60 (8)	p = 0.643#
cLSV	114.99±5.44 (7)	122.53±1.94 (8)	p = 0.355#
BnSTd	120.22±3.89 (7)	122.27±2.60 (8)	p = 0.661
mPVN	140.81±4.35 (7)	145.78±2.15 (8)	p = 0.355
CeA	117.29±6.42 (7)	126.47±3.11 (8)	p = 0.203
VTA	94.72±4.89 (7)	103.43±2.33 (8)	p = 0.117
PAG	90.48±3.96 (7)	92.35±2.74 (8)	p = 0.698
lPAG	92.23±4.21 (7)	93.12±2.53 (9)	p = 0.852

Notes: The sample numbers listed above were the final sample numbers used for data analysis. A complete list of original sample numbers for each brain region can be found in the Methods section. Missing or torn sections led to some regions having fewer samples than others.

^#^ indicates that data failed the assumption of equal variance, and a Kruskal-Wallis ANOVA on Ranks was performed. The p-value for those regions are derived from the Kruskal-Wallis ANOVA on Ranks, while the mean ± SEM were taken from the results of the one-way ANOVA. Abbreviations: rLSD = rostral dorsal LS; rLSV = rostral ventral LS; mLSV = medial ventral LS; cLSV = caudal ventral LS; mPVN = medial PVN; lPVN = lateral PVN; cPVN = caudal PVN; CeA = central amygdala; PAG = periaqueductal gray; lPAG = lateral periaqueductal gray.

## Discussion

The goal of this study was to determine if there were endogenous changes in NT and NT receptor expression in postpartum compared to virgin females. Specifically, we found both up-regulation and down-regulation of NT and NT receptors in postpartum females compared to virgins. The results suggest that NT may contribute to promoting or suppressing particular maternal behaviors, and that NT and NT receptor expression changes are region specific. The qPCR and IHC results are discussed together in the context of the regions in which they were found.

### NT Expression is Increased at the mRNA and Peptide Level in the Postpartum MPOA

The expression of NT and Sort1 were up-regulated in postpartum females in the MPOA, a region which has previously been linked to maternal behaviors such as pup retrieval, nest building, and nursing behavior [2]. In situ hybridization targeting NT mRNA expression has indicated that NT is produced in specific preoptic area nuclei, including the central MPOA and ventral MPOA, both of which are necessary for the production of pup retrieval and nesting behavior [9]. NT production has also been linked to preoptic nuclei that contain estrogen receptor and GAD67 mRNA, which have differential expression in the postpartum period and are linked with maternal behaviors [9], [43]–[46]. In addition, estrogen increases the production of NT via a cAMP pathway, and NT stimulates the release of GABA [47], [48]. Importantly, it has been found that cFos expression resulting from virgin sensitization to pups is found in 1/3 of cells expressing NT mRNA in the MPOA [9]. The relatively large proportion of NT containing cells activated following pup sensitization, as well as the pivotal location of NT and its localization with two other regulators of maternal behaviors, suggests that elevated NT expression in the MPOA of postpartum females may contribute to the expression of maternal behavior.

Immunohistochemical analysis revealed an increase in NT labeling in the MPOA of postpartum females, but no direct evidence exists to indicate if the NT was produced locally or transported to the MPOA for release. However, given the increase in NT gene expression in the MPOA, coupled with the numerous cell bodies labeled for NT mRNA [24], [49], it is possible that NT labeling seen in the MPOA is the result of increased production of NT in that region. The MPOA has reciprocal connections with the LS, central amygdala, and periaqueductal gray, and receives afferent input from the BnST, PVN, and VTA [50], [51] which produce NT. While this study finds elevated synthesis of NT mRNA and NT peptide in the MPOA, additional work is needed to determine where the increased NT is acting.

### Alterations in Ntsr1 mRNA and NT Peptide Expression in the Maternal PVN

In the PVN, Ntsr1 mRNA expression was decreased in postpartum females. The PVN is of interest because it is involved in maternal behaviors and also regulates HPA activity [52]–[54]. Ntsr1 dependent mechanisms also modulate HPA axis activity in the PVN [34], [35]. The down-regulation of Ntsr1 expression in the postpartum PVN could reflect or contribute to attenuation of the HPA axis, which occurs in postpartum females [55].

In contrast to the decreased Ntsr1 mRNA in the PVN, NT peptide labeling in two subregions of the PVN is elevated in postpartum females. Because NT in situ hybridization studies have found relatively few cell bodies in the PVN that contain NT mRNA (Fig. 3B) [24], [49], the elevated NT protein indicates that more NT is being transported into the PVN than is being produced there. The PVN receives afferent input from the LS, medial amygdala, MPOA and LH, but it is unknown which of those send afferent projections to the PVN containing NT [56]–[58]. If increased levels of NT were being released in the PVN, it may also affect the milk ejection reflex, since PVN lesions have been shown to decrease the magnitude and frequency of milk released during suckling [59]. The PVN is one of two key sites for production of oxytocin, which has been linked to maternal care in numerous studies [8], [12], [60] and NT has been shown to affect the release of oxytocin [10].

It is possible that the down-regulation of Ntsr1 in the PVN of postpartum females is caused by elevated release of NT. Exposure to an NT analog leads to destabilization of Ntsr1 mRNA in mouse neuroblastoma and HT-29 cells [61]–[63]. However, to our knowledge, no in vivo studies have been conducted to examine the effects of NT on Ntsr1 mRNA activity. At this point, we cannot conclude that elevated NT IHC corresponds to elevated NT release. Additional studies will be useful for clarifying possible NT release and Ntsr1 interactions.

### NT mRNA or Peptide Expression is Differentially Regulated in the LS, BnSTd, or LH

The down-regulation of NT mRNA in the LS of postpartum females is consistent with past studies which showed that lowering of NT activity can be linked to elevated offspring protection [13], [64]. The LS contributes to aggressive and certain stress related behaviors [65] and pharmacological manipulations in the LS modulate the expression of offspring protection in postpartum females [66]–[68]. The LS may participate in the stress response of postpartum females and the lowering of NT in the LS could be a critical event for the emergence of offspring protection. It is of interest that the elevation of NT activity in one region, such as the MPOA, could possibly promote certain core maternal behaviors, while decreasing activity in another region, such as the LS, could support a different type of maternal care that is linked to pup defense.

NT gene expression was up-regulated in the BnSTd, a region that coordinates the emotional and behavioral responses to stress [69]. Electrical stimulation of different BnST subregions can affect circulating plasma corticosterone levels, and projections between the BnST and the PVN may modulate the HPA axis [56], [70]–[72]. The increased NT gene expression could act locally or be transported to other regions for release. One possibility is that elevated NT mRNA in the BnSTd could lead to the elevated NT IHC levels observed in either the PVN or MPOA, but this needs to be tested directly. NT acting in the BnSTd may also affect maternal behaviors, since oxytocin activity is altered in the BnST of lactating females [73], [74].

Immunohistochemical results indicate that NT protein labeling is elevated in the LH of postpartum females. Few cell bodies were labeled in the LH, and the staining was almost exclusively of fibers. It is difficult to ascertain whether the increased labeling is due to increased expression of NT in neurons of the LH or the medial forebrain bundle (mfb). Cell bodies containing NT have been found in the region of the LH/mfb [22], [23], and the LH receives neurotensinergic input from the LS, BnST, MPOA, PVN and CeA [75]. However, fibers projecting through the mfb may also be carrying NT to other regions, or transporting it from other regions to the LH [76]. Lesioning different parts of the LH have been shown to affect maternal behaviors [77]. Hypocretin, which is produced almost exclusively in the LH, alters levels of pup licking and grooming and offspring projection when injected into postpartum females [78]–[80]. Though we are unable to determine if NT peptide expression is different in fibers of the LH or mfb, the change is taking place in an area associated with maternal behavior and interconnected within the maternal behavior circuitry.

### Nonsignificant Decrease in NT mRNA in the VTA of Postpartum Females

In the VTA, there was a statistically nonsignificant decrease in NT mRNA levels in maternal females. The sample size used for analysis was smaller relative to some other regions tested (e.g. NAcc, LS, LH) and may have reached significance if a larger number of animals were used. Although this result is not significant, it may still be biologically relevant given the extensive interactions between dopamine and neurotensin within the VTA [29], [81], and the involvement of dopamine in some maternal behaviors [31], [33]. However, additional studies would be required to evaluate possible changes in NT and interactions between NT and dopamine in the VTA in postpartum females to address this possibility.

### Possible Mechanisms for Altering NT and NT Receptor Expression in Postpartum Females

The mechanisms which lead to changes in NT and NT receptor expression in the postpartum female are currently unknown. Estrogen increases NT expression and the promoter region of NT contains AP-1, cAMP response element (CRE), and glucocorticoid response element sites [47], [82]. Ntsr1 contains Sp1 and AP-2 binding sites in its promoter region [83], and Ntsr2 contains CREB, Oct-2, Ikarous-2 and GATA-2 binding elements [84]. Following pro-neurotensin/neuromedin N translation, the proprotein is packaged into dense core vesicles, cleaved by protein convertases into the tridecapeptide NT and the hexapeptide neuromedin N, and transported to axon terminals for release [85], [86]. Following NT release at the synapse, NT is either bound to a receptor or degraded via endopeptidases [87]. The G-protein coupled receptors, Ntsr1 and Ntsr2, internalize following agonist binding, and are then degraded or recycled to the cell surface [88]. Sort1 is found primarily within the cell [21], and a model has been proposed suggesting that Sort1 may aid in the transportation of receptor bound NT to the perinuclear region [63]. We found a significant up-regulation of Sort1 mRNA in the postpartum MPOA, which may be contributing to intracellular transportation of NT. However, to our knowledge, experiments to test the validity of this model have yet to be conducted, and further studies analyzing the interactions between Sort1 and NT bound receptors in vivo are necessary.

The expression of Ntsr2 did not change between virgin and postpartum females in any region tested, suggesting that Ntsr2 may not participate in the appearance of maternal behaviors. Knockout studies have been conducted for NT, Ntsr1 and Ntsr2, but we did not utilize these mice for the current study because of potential compensatory effects following the elimination of a gene [37], [89]–[92]. Instead, we utilized outbred mice to evaluate endogenous changes in NT and NT receptor expression, and we expect that our findings would be broadly applicable to rodents or other species, though this is not known. Subsequent studies utilizing site specific injections of NT siRNA may determine specifically what behaviors NT and NT receptor expression are associated with.

The virgin females used in this study differed from the postpartum females in several aspects. Virgin females were housed with female conspecifics while maternal females were housed with males and underwent mating. Both virgin and maternal females were isolated following mating, but maternal females were then exposed to pups for approximately one week while the virgin females remained isolated. The maternal females also underwent pregnancy, parturition, and lactation while the virgin females did not. The justification for using virgins as a control is that they have not experienced events which contribute to the formation of the maternal brain, while the maternal group are fully functioning mothers. This paradigm allows us to determine what are the key changes in the maternal brain before parsing out what specific events contribute to alterations in gene expression. The goal of this study was to determine if endogenous levels of NT and NT receptors change during the transition from a virgin to a postpartum state, which provides a basis for subsequent studies to analyze NT and NT receptor expression in virgins exposed to pups, pregnant females, or postpartum females deprived of pups. Further examinations will be able to elucidate the time course of changes in NT and NT receptor levels, and what behaviors NT signaling is associated with.

This research furthers past studies linking NT to maternal care and studies that indirectly identify neurotensinergic signaling alterations in the postpartum brain, although the changes appear to be dynamic and vary across specific brain regions. The nature of the changes in NT signaling in the postpartum brain may reflect the individual role each brain region, or subset of brain regions, plays in the broader maternal behavior circuitry.

## Conclusions

This study demonstrated for the first time that endogenous NT and NT receptor expression changes between the virgin and postpartum period. We were able to determine what maternally linked brain regions exhibited changes in NT and NT receptor expression, and suggest where altered levels of NT peptide are produced and released. The altered signaling of NT in the postpartum brain supports previous research indicating its involvement in the suppression of certain maternal behaviors, and suggests its involvement in the appearance of others. Future studies probing NT activity in the appearance of other maternal behaviors, specifically within the MPOA, are warranted.

## Methods

### Ethics Statement

All procedures followed the guidelines of the National Institutes of Health Guide for the Care and Use of Laboratory Animals and were approved by the University of Wisconsin Animal Care and Use Committee (protocol # L00422-0-06-10).

### Animals

Age-matched (∼70 days at time of dissection) female outbred hsd:ICR (Harlan, Madison, WI) mice were used for all experiments. One half of the females were housed with other females for 10–14 days while the other half were housed with a breeder male (hsd:ICR strain). When males were removed, all females were individually housed for the remainder of the study. This housing strategy provided a similar social environment for all females and minimized the possibility of isolation induced stress [93], [94] that could affect gene expression [95]. Virgin females were used as a control because they are naive to the experiences that construct the maternal brain, and have been used successfully in conjunction with maternal females to study anxiety related behaviors [96], the role of MAPK in anxiety related behaviors [97], oxytocin receptor changes [98], and cell proliferation [99]. Mice were housed in polypropylene cages with nestlets that were changed weekly prior to parturition. Following parturition, cages remained unchanged for the duration of the study. On postpartum day 0, litters were culled to 11 pups and females with less than 9 pup were omitted from the study. All females were provided with ad libitum breeder chow (Harlan, Madison WI) and water. Animals were housed on a 12:12 light/dark cycle with lights on at 6:00 CST for the qPCR study, and on a 14:10 light/dark cycle with lights on at 6:00 CST for the IHC study.

### Tissue Collection and cDNA Preparation for qPCR

On postpartum day 7, brains were removed from lactating females and age-matched virgin females between 9:00 and 12:00 CST and estrous states were determined using protocols previously described [46], [100]. Brains were sliced on a cryostat (Leica, CM1850, Bannockburn, IL, USA) at a thickness of 200 µm and mounted onto slides. Regions of interest were removed using a micropunch technique using the Brain Punch Set (Stoelting, Wood Dale, IL, USA) under a dissection microscope and frozen at −80°C. The bregma coordinates for regions collected were as follows: NAcc (1.70 mm to 0.98 mm), LS (1.10 mm to 0.14 mm), BnSTd (0.26 mm to 0.02 mm), MPOA (0.26 mm to −0.10 mm), PVN (−0.58 mm to −0.94 mm), LH (−0.70 mm to −1.06 mm), BLA/CeA (−0.82 mm to −1.70 mm), and VTA (−2.92 mm to −3.80 mm). Samples for the NAcc, LS, BnSTd, PVN, LH, and BLA/CeA were collected from 14 virgin and 14 postpartum females. Two individual samples were pooled for the BnSTd and PVN for a total of 7 virgin and 7 postpartum samples. 12 virgin and 12 postpartum females were used from the original group for the NAcc, LS, LH, and BLA/CeA and the remaining unused samples were used to make standards for qPCR. A separate group of females were used for the MPOA and VTA, for a total of 7 virgin and 8 postpartum samples. All virgin females used for qPCR were in diestrous. Total RNA was extracted using the Aurum Total RNA Fatty and Fibrous tissue kit (Bio-Rad, Hercules, CA) with some minor alterations to the manufacturer's protocol. Briefly, two low stringency washes were added just before RNA elution for smaller brain regions with lower concentrations of RNA (BnSTd, LH, MPOA, and PVN), and total RNA for all regions was eluted with 30 µL nuclease free water heated to 70°C instead of the elution solution provided by the manufacturer. Samples were randomly selected from each region to test RNA integrity using Agilent RNA 6000 Nano Chips or Pico Chips with the Agilent Bioanalyzer 2100 (Agilent Technologies, Palo Alto, CA). Regions with fewer total samples (BnSTd, MPOA, PVN, VTA) had 6 samples tested, and regions with more samples (NAcc, LS, LH, BLA/CeA) had 12 samples tested. RNA concentration was determined using a NanoDrop 1000 and 2000 spectrophotometer (Thermo Scientific, Wilmington, DE). 100 ng of RNA from each sample was reverse transcribed using a SuperScript III First Strand Synthesis for RT-PCR kit (Invitrogen, Carlsbad, CA) in an Eppendorf MasterCycler Personal PCR machine (Eppendorf, Hamburg, Germany). The cDNA was diluted and the final concentration determined using a NanoDrop 1000 or 2000 spectrophotometer to ensure there was no significant difference in cDNA concentration between lactating and virgin females.

### RT-qPCR and Data Analysis

The cDNA was amplified in a StepOnePlus Real Time PCR machine (Applied Biosystems, Foster City, CA) using a protocol previously described [46] and primers designed using NCBI Primer Blast (Table 3). Dissociation curves were generated for all reactions to ensure primer specificity and a standard curve was generated to determine the reaction efficiency using StepOnePlus software. The baseline for all reactions done with NT primers was adjusted to represent the mean fluorescence between cycles 3 and 15. All other reactions used an autocorrected baseline. The relative expression software tool REST 2009 was used to determine the relative expression of genes in lactating females compared to virgin females. All genes were normalized to two reference genes (Ywhaz, Ppia, Ywhah or Sdha) for each region except for the PVN where only Sdha was used. The reference genes used have been previously shown to be stably expressed in mouse brain or showed no differential expression in previous microarrays performed between lactating and virgin females [101]–[103]. Further, if a reference gene in a particular region trended toward a significant difference in expression between the two groups, then it was excluded and more stable reference genes for that region were used.

**Table 3:** Primers for genes of interest and reference genes used for real-time qPCR.

Gene Symbol	Gene Name	NCBI Accession Number	Annealing Temp		Primer Sequence
NT	Neurotensin	NM_024435.2	61°C	Forward	5′-GTG TGG ACC TGC TTG TCA GA-3′
				Reverse	5′-TCA TGC ATG TCT CCT GCT TC-3′
Ntsr1	Neurotensin receptor 1	NM_018766.2	57°C	Forward	5′-TCT GAT GTT GGA CTT GGG TTC-3′
				Reverse	5′-AGT GCT ATG GTA TCT GCT GG-3′
Ntsr2	Neurotensin receptor 2	NM_008747.2	57°C	Forward	5′-TCT CTC AGT TCC CTG TGT GG-3′
				Reverse	5′-AGC AGC CAT TGT TTG TTC TC-3′
Sort1	Sortilin receptor 1/Neurotensin receptor 3	NM_019972.2	57°C	Forward	5′-TTC CCA GAC TAT CCT CAC CC-3′
				Reverse	5′-TAT TGA CCA CAC ACG GCA TC-3′
Ppia	Peptidylprolyl isomerase A	NM_008907.1	58°C	Forward	5′-TGC TGG ACC AAA CAC AAA CG-3′
				Reverse	5′-GCC TTC TTT CAC CTT CCC AAA-3′
Sdha	Succinate dehydrogenase complex, subunit A	NM_023281.1	58°C	Forward	5′-CCG CTC CTA CTG ATG AAA CC-3′
				Reverse	5′-GCG CAA CTC AAT CCC TTA C-3′
Ywhah	Tyrosine 3 monooxygenase/tryptophan 5 monooxygenase activation protein, eta	NM_011738.2	57°C	Forward	5′-GAC AAGA GCT GAA TGA ACC AC-3′
				Reverse	5′-TAA CCC TCC AAG AAG ATC GC-3′
Ywhaz	Tyrosine 3-monooxygenase/tryptophan 5 monooxygenase active protein, zeta polypeptide	NM_001253805.1	58°C	Forward	5′-TCC TTA TTC CCT CTT GGC AG-3′
				Reverse	5′-ATG GAA GCT ACA TTA GCG GTT T-3′

### NT Antibody Specificity

**Figure 5:**
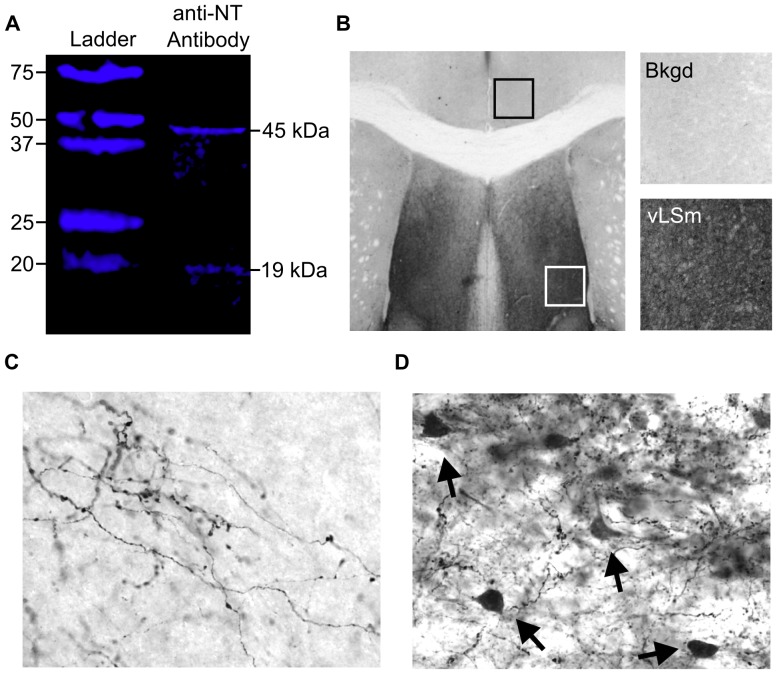
Images from a near infrared western blot and immunohistochemical staining using an anti-NT antibody. (A) Bands were visualized at approximately 19 kDa, which corresponds with the molecular weight of the neurotensin/neuromedin N precursor protein, and at approximately 45 kDa. The identity of the protein weighing 45 kDa is unknown. (B) Example of the normalization method used to control for differences in background labeling. Background staining was relatively low across all sections analyzed (black square and top right panel), and was subtracted from the densitometric mean of regions of interest, such as the vLSM (white square and bottom right panel) in order to normalize brain sections analyzed. (C–D) NT staining was found in fibers and cell bodies (D, black arrows), which was expected. Regions labeled for NT were consistent with past immunohistochemical studies, suggesting that the antibody is specific to NT.

Rabbit anti-NT primary antibodies (Catalog # 20072, ImmunoStar Inc, Hudson, WI) were used for IHC. The antibodies have been used in multiple studies [104]–[106] and incubation with synthetic NT has been shown to eliminate immunohistochemical staining [106]. We confirmed this observation by performing a peptide competition assay using synthetic NT (Sigma Aldrich) and a protocol similar to those previously used (data not shown) [106]. We also evaluated antibody specificity using a near-infrared western blot. Micropunched tissue previously collected from the LS, LH, NAcc, and BLA/CeA was combined and protein was extracted, measured, and a western blot was performed using a protocol similar to one previously described [46]. Briefly, an IRDye 680/800 nm Protein Marker (LI-COR Biosciences, Lincoln, NE) and 20 µg of protein in sample buffer was added to a 10–20% Mini Protean Tris-Tricine Precast Gel (Bio-Rad). Protein was transferred to a PVDF membrane, then washed in 0.1 M tris buffered saline with 0.05% Tween 20 (TBST), incubated in Odyssey Blocking Buffer (LI-COR) for one hour, then incubated overnight at 4°C in a 1∶50 dilution of NT primary antibody with TBST and 5% blocking buffer. The membrane was then incubated in Odyssey donkey anti-rabbit Infrared Dye labeled secondary antibody (LI-COR) before being washed with TBST and 0.1 M tris buffered saline (TBS). Bands were detected using the Odyssey Fc in the 800 nm channel for the protein marker and the anti-NT antibody. The western blot revealed two bands weighing approximately 19 kDa and 45 kDa (Fig. 5A). The 19 kDa band corresponds with the molecular weight of the neurotensin/neuromedin N precursor protein [82], [107], however the identity of the 45 kDa protein is unknown. The amino acid sequence targeted by the NT antibody was entered into the NCBI Protein BLAST. Of the top 10 proteins with sequence similarity to NT, only one, solute carrier family 45, member 2 (Slc45a2) was found to have an isoform with a molecular weight close to 45 kDa. However, expression of Slc45a2 is widespread across the CNS [49], and anti-NT labeling in tissue is region specific and highly consistent with previous reports of NT distribution, including the NT GFP construct from the Rockefeller Institute [22], [23]. Therefore, it is not likely the antibodies are recognizing the Slc45a2 isoform in the tissue.

### Tissue Collection and NT Immunohistochemistry (IHC)

On postpartum day 6–7, lactating females and age matched virgin females were anesthetized with isoflourane and decapitated. Brains were fixed overnight in 5% acrolein and phosphate buffered saline solution (PBS) at 4°C, then transferred to a 30% sucrose solution in PBS for 2 days. Before slicing, brains were briefly frozen on dry ice, then sliced on a cryostat to a 40 µm thickness. All slices were stored in cryoprotectant at −20°C until IHC analysis.

The IHC procedure used in this study is similar to ones previously described [15], [108]. In brief, sections were first washed in 1.8% hydrogen peroxide for 10 minutes and then 0.5% sodium borohydride for 30 minutes before being washed in PBS with 0.3% triton-x (PBS-X). Sections were then blocked with 5% normal goat serum (Vector Labs, Burlingame, CA) in PBS-X, then incubated in a 1∶12,000 dilution of anti-NT primary antibody for two days at 4°C. After incubation, slices were exposed to a biotinylated goat anti-rabbit secondary antibody (Vector Labs) for 90 minutes, an avidin biotin complex with peroxidase for 1 hour (Vector Labs), then 3, 3′-diaminobenzidine (Fisher Scientific) for 5 minutes before being washed with PBS and mounted onto slides. The slides were dehydrated in ethanol and xylene and coverslipped with permount before staining was analyzed.

### Analysis of NT IHC Labeling

An Axioskop Zeiss light microscope and an Axiocam Zeiss digital camera interfaced with the computer were used to measure the density of NT labeling in our regions of interest. The majority of labeling was found in fibers with very few cell bodies labeled (Fig. 5C–D), therefore no cell counting was conducted. The mean density of staining was determined for each region, and one section was counted for each brain region for all animals. The rostral dorsal portion of LS (rLSD), and all PVN subregions analyzed used a magnification of 20×. All other regions examined used a magnification of 10×. The bregma coordinates used for regions of interest were based on the Allen Mouse Brain Atlas (http://mouse.brain-map.org), and are as follows: NAcc and medial ventral LS (mLSV) (0.845 mm); rLSD and rostral ventral LS (rLSV) (1.045 mm); caudal ventral LS (cLSV) and BnSTd (0.145 mm); MPOA (−0.08 mm); lPVN and medial PVN (mPVN) (−0.48 mm); cPVN, LH, and CeA (−1.055 mm); VTA and periaqueductal gray (PAG) (2.85 mm); and the lateral PAG (lPAG) (−3.68 mm). Frame sizes used to measure the mean density for each brain region are as follows: 300×300 frame size for mPVN, lPVN, and the PAG; 400×450 for BnSTd and VTA; 530×400 for the lPAG; 512×512 for NAcc, rLSV, mLSV, cLSV, MPOA, LH, and CeA; 530×710 for rLSD; and 710×530 for cPVN. To make certain that the mean density was measured consistently for all animals, a number of steps were taken to normalize the sections: 1) during IHC, all sections were run in one batch, 2) slides were coded and all microscopy work was done by an individual blind to the experimental conditions, 3) backgrounds were normalized for each section by adjusting light levels, and 4) the mean background density was subtracted from the mean density of labeling for each region of interest. Subtracting out the background from each region of interest accounts for any variation in labeling that was not removed after the light levels were adjusted (Fig. 5B). A one-way analysis of variance (ANOVA) was run for each region between the two groups using SigmaPlot (Systat Software, San Jose, CA). In the event that the assumption of equal variance or normality was not met, a Kruskal-Wallis one way ANOVA on ranks was used. The virgin female group consisted of 4 diestrous and 3 estrous females (except for rLSD, which had 4 diestrous and 2 estrous females), but one-way ANOVA results indicated that there was no significant effect of estrous state on gene expression.
